# Continuous Monitoring of Mental Load During Virtual Simulator Training for Laparoscopic Surgery Reflects Laparoscopic Dexterity: A Comparative Study Using a Novel Wireless Device

**DOI:** 10.3389/fnins.2021.694010

**Published:** 2022-01-20

**Authors:** Neta B. Maimon, Maxim Bez, Denis Drobot, Lior Molcho, Nathan Intrator, Eli Kakiashvilli, Amitai Bickel

**Affiliations:** ^1^The School of Psychological Sciences, Tel Aviv University, Tel Aviv, Israel; ^2^Neurosteer Ltd, Herzliya, Israel; ^3^Medical Corps, Israel Defense Forces, Ramat Gan, Israel; ^4^Faculty of Medicine in the Galilee, Bar-Ilan University, Safed, Israel; ^5^Department of Surgery A, Galilee Medical Center, Nahariyya, Israel; ^6^Blavatnik School of Computer Science, Tel Aviv University, Tel Aviv, Israel

**Keywords:** cognitive load, surgical simulator training, EEG biomarker, laparoscopic operations, brain assessment, mental load assessment, machine learning

## Abstract

**Introduction:**

Cognitive Load Theory (CLT) relates to the efficiency with which individuals manipulate the limited capacity of working memory load. Repeated training generally results in individual performance increase and cognitive load decrease, as measured by both behavioral and neuroimaging methods. One of the known biomarkers for cognitive load is frontal theta band, measured by an EEG. Simulation-based training is an effective tool for acquiring practical skills, specifically to train new surgeons in a controlled and hazard-free environment. Measuring the cognitive load of young surgeons undergoing such training can help to determine whether they are ready to take part in a real surgery. In this study, we measured the performance of medical students and interns in a surgery simulator, while their brain activity was monitored by a single-channel EEG.

**Methods:**

A total of 38 medical students and interns were divided into three groups and underwent three experiments examining their behavioral performances. The participants were performing a task while being monitored by the Simbionix LAP MENTOR*™*. Their brain activity was simultaneously measured using a single-channel EEG with novel signal processing (Aurora by Neurosteer^®^). Each experiment included three trials of a simulator task performed with laparoscopic hands. The time retention between the tasks was different in each experiment, in order to examine changes in performance and cognitive load biomarkers that occurred during the task or as a result of nighttime sleep consolidation.

**Results:**

The participants’ behavioral performance improved with trial repetition in all three experiments. In Experiments 1 and 2, delta band and the novel VC9 biomarker (previously shown to correlate with cognitive load) exhibited a significant decrease in activity with trial repetition. Additionally, delta, VC9, and, to some extent, theta activity decreased with better individual performance.

**Discussion:**

In correspondence with previous research, EEG markers delta, VC9, and theta (partially) decreased with lower cognitive load and higher performance; the novel biomarker, VC9, showed higher sensitivity to lower cognitive load levels. Together, these measurements may be used for the neuroimaging assessment of cognitive load while performing simulator laparoscopic tasks. This can potentially be expanded to evaluate the efficacy of different medical simulations to provide more efficient training to medical staff and measure cognitive and mental loads in real laparoscopic surgeries.

## Introduction

Medical training of surgical interns in recent years utilizes digital simulators as an effective tool for acquiring practical skills in a controlled and hazard-free environment. These simulators help train interns and provide their performance outputs for each task, thus supporting the learning process. The surgery simulators offer practice of various tasks and present an evaluation of the performance *via* behavioral measurements (i.e., performance accuracy and performance length, etc.). Nevertheless, the simulator output measurements have two main drawbacks. First, althoughthe behavioral performance serves as a good indication of laparoscopic dexterity, it does not give an indication regarding the student’s cognitive state and, specifically, his or her cognitive load level during the task performance. Assessing cognitive load and stress levels directly during the surgery simulation is essential to evaluate the intern’s ability to engage in real-life operations. Second, the behavioral performance is measured while completing a task under the surgery simulator. Therefore, it does not allow extraction of similar measurements during laparoscopic procedures inside the operating room. To address these points, we designed three experiments in which medical students and interns perform laparoscopic tasks under a surgery simulator, while recording their brain activity with a wearable single-channel EEG headset. The experiments measured behavioral performance, EEG bands that are known to correlate to cognitive load, and a novel machine-learning-based cognitive load biomarker. In the following paragraphs, we will review the literature regarding cognitive load, EEG biomarkers of cognitive load, medical simulators, and previous measurements of cognitive load during simulator tasks.

### Cognitive Load

Any acquirement of a new skill or knowledge must be passed through working memory (WM) before being transferred into long-term memory (LTM). According to Cognitive Load Theory (CLT, Miller, 1956), WM resources, unlike LTM resources, are limited in their capacity for processing or holding new information. However, when performing a complex task, the new information elements are being processed simultaneously and not iterated, so their interactions cause a much higher WM load. According to CLT, optimizing learning processes may be achieved by constructing automated schemas in which the WM load is reduced (Jolles et al., 2013). The amount of cognitive load used is a good predictor of the learning process during a task performance, and continuous measurement is of particular importance (Van Merriënboer and Sweller, 2005; Constantinidis and Klingberg, 2016). For instance, practicing the same task repeatedly will cause some of the interacting information elements to be stored together in a schematic form in LTM, so that they can be extracted and manipulated in WM as single-information elements (Schneider and Shiffrin, 1977).

### Cognitive Load Biomarkers

Several methods were previously described and validated as measures of WM and cognitive load. Traditionally, subjective self-rating scales were proven as reliable assessment tools across several studies (Paas et al., 2003). However, as this tool can only be recorded crudely and retrospectively, objective assessment methods with a real-time indication of WM are in demand. By using such an approach, the activity can be broken down into different components that reflect different stages of complex simulations and be used to evaluate the efficacy of each training element. Several biological methods have been reported to successfully assess WM, such as pupil size (Van Gerven et al., 2004), tracked eye movement (Van Gog and Scheiter, 2010), salivary cortisol levels (Bong et al., 2010), and functional magnetic resonance imaging (fMRI) (Van Dillen et al., 2009).

Using electroencephalography (EEG), studies repeatedly show that the theta band (4–7 Hz) measured by frontal electrodes increases with higher cognitive load and task difficulty (Scheeringa et al., 2009; Antonenko et al., 2010). Multiple studies confirmed this correspondence using a variety of cognitive tasks, including the *n*-back (Gevins et al., 1997), operation span task (Scharinger et al., 2017), visuospatial WM tasks (Bastiaansen et al., 2002), and simulated real-life experiences such as in driving (Di Flumeri et al., 2018) or flight simulators (Dussault et al., 2005).

In addition to theta, delta oscillation (0.5–4 Hz) was also found to increase with higher cognitive load and/or task difficulty such as during reading tasks (Zarjam et al., 2011) and multiple-choice reaction tasks (Schapkin et al., 2020), and to increase with higher vigilance during low-cognitive-load attention tasks (Kim et al., 2017). It has been suggested that delta activity is an indicator of internal attention, and therefore increases while undergoing mental tasks (Harmony et al., 1996). Delta activity was also associated with interference inhibition processes, which occur in order to modulate sensory afferences, which in turn increase internal concentration (Harmony, 2013).

### Medical Simulations

Medical simulations are a common and widespread tool for medical education (Gurusamy et al., 2009). Medical simulations can emulate common scenarios in clinical practice, and through interactive interplay and hands-on teaching, they can improve the effectiveness and quality of teaching of healthcare professionals (Kunkler, 2006). These simulations can be particularly beneficial for surgical staff, as they allow residents to practice and perfect complex procedures, ensuring they have enough experience and practice before real patient contact (Thompson et al., 2014). As laparoscopic surgeries demand unique eye–hand coordination and are performed while the surgeon indirectly observes the intra-abdominal contents without tactile sensation but through a camera view, these surgeons are ideal candidates for virtual reality (VR) simulators. Indeed, these simulators have been shown to greatly improve surgical operating skills and reduce operating time (Gallagher et al., 2005; Larsen et al., 2009; Gauger et al., 2010; Yiannakopoulou et al., 2015; Tucker et al., 2017).

Recently, a few studies made the connection between CLT and medical training using surgery simulators (Andersen et al., 2016, 2018; Frederiksen et al., 2020). In these studies, participants completed laparoscopic tasks *via* VR simulators, while their WM loads were estimated using a secondary behavioral task (e.g., reaction times in response to auditory stimuli). They found that different interventions with medical VR training may modulate WM load. For example, Frederiksen et al. (2020) showed that immersive VR results show higher cognitive load than the conventional VR training, and Andersen et al. (2018) demonstrated that additional VR training sessions may reduce cognitive load and increase the performance of dissection training. However, these studies evaluated cognitive load to assess the effect of an external manipulation, rather than to understand the neurophysiological mechanism underlying the simulation process itself. None of the studies recorded an objective physiological WM load biomarker such as pre-frontal cortex activity or frontal theta oscillation during the training itself. Such measurements would not only exhibit a continuous level of cognitive load, but, if found reliable, could later be measured in real scenarios. Lower levels of WM load, along with efficient performance, may indicate the readiness levels of surgeons. Additionally, a wearable, portable device may enable real-time monitoring of WM load levels inside the operating room, reflecting a surgeon’s abilities and laparoscopic dexterity.

### The Present Study

The aim of this study was to explore the neural mechanism that underlies surgery simulation training. Specifically, our aim was to test the relationship between cognitive load, skill acquisition, and the activity levels of different brain oscillations. We aimed to track cognitive load neuro-markers using an EEG device that will enable hand mobility while performing surgical simulator tasks. Importantly, we wanted to test medical students and interns who, on the one hand, have great motivation and motor/cognitive abilities to perform such laparoscopic tasks and, on the other, have no previous hands-on experience, neither in surgery simulators nor with real-life patients. Finally, we intended to compare “online gains”—improvements in performance that occur while undergoing the task—to “offline gains,” the improvements in skill acquisition preceding a consolidation during nighttime sleep between task trainings, without further practice (Issenberg et al., 2011; Fraser et al., 2015; Lugassy et al., 2018). Notwithstanding, frontal theta and other known cognitive load biomarkers were mostly used to measure WM load during task completion itself and not to evaluate the load differences between tasks’ sessions.

To meet these goals, we divided 38 medical students and interns into three experiments. Each experiment included three trials of the same short laparoscopic task administered by a surgical simulator (Simbionix LAP MENTOR*™*). While performing the tasks, participants’ brain activity was recorded using a wearable single-channel EEG device (Aurora by Neurosteer^®^ Inc), from which we continuously measured frontal brain oscillations (i.e., the theta and delta bands) throughout the simulation task. In each experiment, the participants repeated the same task three times, thus allowing them to obtain the new skill and decrease their cognitive load levels.

In addition to known brain oscillations, Neurosteer^®^ also provided us with a machine-learning-based cognitive load biomarker (i.e., VC9). VC9 activity was previously shown to increase with cognitive load in the standard and most common WM tasks (i.e., the *n*-back task, Maimon et al., 2020), auditory recognition and classification tasks (Molcho et al., 2021), and interruptions paradigm (Bolton et al., 2021). Specifically, Maimon et al. (2021) showed that the VC9 biomarker is more sensitive to the finer differences between cognitive loads. During the *n*-back task, while both VC9 activity and theta and delta oscillations increased with higher cognitive loads, the VC9 biomarker exhibited higher sensitivity than the theta and delta oscillations to the lower levels of cognitive load. These subtler differences are particularly crucial for the present study’s purposes and experimental design. With each task repetition, participants’ cognitive load levels descended, eventually reaching relatively low levels of cognitive load. Therefore, the ability to detect finer differences between the low cognitive loads is the most critical for estimating participants’ readiness to go into the operating room.

**FIGURE 1:**
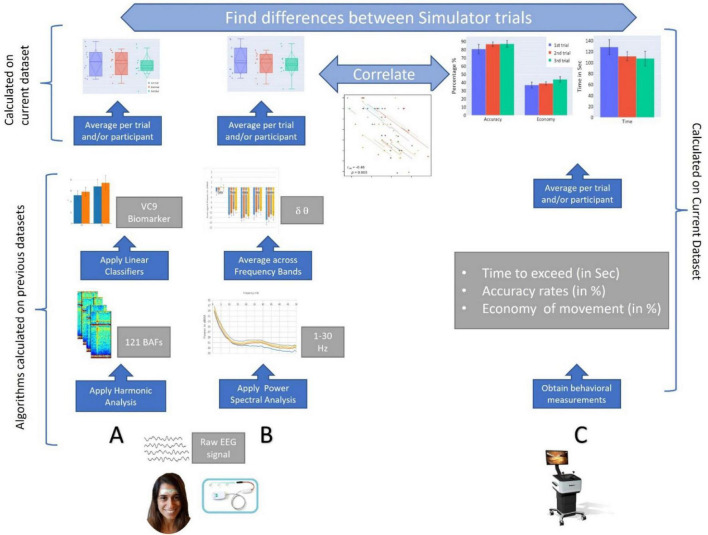
A graphical representation of the study. **(A)** EEG data are recorded *via* a single channel using a three-electrode forehead patch by an Aurora EEG system while participants perform the simulator task. Data are pre-processed by a predefined set of wavelet packet atoms to produce 121 Brain Activity Features (BAFs). The BAFs are passed through linear models, which were pretrained on external data, to form a higher-level biomarker, VC9. Then, VC9 activity is averaged per trial and participant to find differences between simulator trials. **(B)** From the raw EEG data, power spectral density analysis is applied to find energy representation (in dB) produced for 1–50 Hz. The power density per Hz is averaged over frequency bands. Theta and delta activity are averaged per trial and participant to find differences between simulator trials. **(C)** Behavioral data are obtained from the Simbionix LAP MENTOR*™* (i.e., time, accuracy, and economy of movement). These are averaged per trial and participant to find differences between simulator trials. Finally, correlations are calculated between EEG features (i.e., VC9, theta, and delta bands) and behavioral performance (time, accuracy, and economy of movement).

Thus, our hypotheses were as follows: (1) Behavioral performance will improve with repetition of the surgery simulator task. (2) This improvement of behavioral performance with task repetition will result in the decrease of cognitive load that will modulate the EEG features (i.e., theta, delta, and VC9 will decrease with task repetition). (3) EEG features will correlate to some extent with higher individual performance (i.e., decrease with better individual performance), reflecting the reduced need for cognitive resources together with improving laparoscopic dexterity. (4) “Offline gains” will be present following procedural memory consolidation during nighttime sleep. Probing these hypotheses will help reveal new and objective information regarding the efficacy of simulation-based training. For a graphical representation of the data analysis applied in the present study, see Figure 1.

## Experiment 1

### Materials and Methods

#### Sample Size Selection

To estimate the sample size required to observe a significant effect of simulator trial repetition in the present study, we conducted an *a priori* sample size analysis with G*Power software (Erdfelder et al., 1996). We based this analysis on the study by Andersen et al. (2016). Their study examined 18 novice medical students who underwent VR simulation training while their WM loads were examined using a secondary response time (RT) task. The experimental design included two groups: a control group (*n* = 9), which received standard instructions, and an intervention group (*n* = 9), which received CLT-based instructions. Similar to our present study, both groups in the Andersen et al. (2016) study underwent two post-training virtual procedures. Therefore, we based our power analysis on the within-group comparison of RTs between the first and second simulator trials of the control group. Moreover, Andersen et al. (2016) inserted these multiple within-participants fix effects into mixed linear models design, which was applied in the current study, as well; they are therefore most suitable for comparison. We conducted this analysis with a desired alpha of 0.05, and a power of 0.80, and calculated effect size from the control group’s average RTs and standard deviation (SD) difference between the two simulator trials. The output of the analysis software was that the minimum required sample size was nine participants.

#### Participants

Nineteen participants (68% females, mean age 28, age range 25–36) were enrolled in the first experiment. All participants were healthy medical interns who had completed 6 years of medical studies, never participated in laparoscopic surgery, and had no prior experience using a surgical simulator. Ethical approval for this study was granted by the Galilee Medical Center Institutional Review Board.

#### Electroencephalography Device

The EEG signal acquisition system included a three-electrode patch attached to the subject’s forehead (Aurora by Neurosteer^®^., Herzliya, Israel). The medical-grade electrode patch included dry gel for optimal signal transduction. The electrodes were located at Fp1 and Fp2, with a reference electrode at Fpz. The EEG signal was amplified by a factor of 100 and sampled at 500 Hz. Signal processing was performed automatically by Neurosteer^®^ in their cloud (see section “Signal Processing” below and Supplementary Appendix A). We were therefore provided with a sample per second of activity of the brain oscillations (i.e., delta, theta, alpha, beta, and gamma) and the VC9 biomarker.

#### Signal Processing

Full technical specifications regarding the signal analysis are provided in Supplementary Appendix A. In brief, the signal-processing algorithm interprets the EEG data using a time/frequency wavelet-packet analysis, instead of the commonly used spectral analysis. The Neurosteer^®^ signal-processing algorithm interprets the EEG data using a time/frequency wavelet-packet analysis, creating a presentation of 121 brain activity features (BAFs) composed of the fundamental frequencies and their high harmonics.

To demonstrate this process, let *g* and *h* be a set of biorthogonal quadrature filters created from the filters G and H, respectively. These are convolution-decimation operators, where, in a simple Haar wavelet, *g* is a set of averages and *h* is a set of differences.

Let ψ_1_ be the mother wavelet associated with the filters *s* ∈ *H*, and *d* ∈ *G*. Then, the collection of wavelet packets ψ_*n*_, is given by:


(1) $$\psi_{2n}=H\psi_{n};\psi_{2n}\left(t\right)=\sqrt{2}\sum_{j\in Z}\text{s}\left(j\right)\psi_{n}\left(2t-j\right),$$

(2) $$\psi_{2n+1}=G\psi_{n};\psi_{2n+1}\left(t\right)=\sqrt{2}\sum_{j\in Z}\text{d}\left(j\right)\psi_{n}\left(2t-j\right).$$

The recursive form provides a natural arrangement in the form of a binary tree. The functions ψ_*n*_ have a fixed scale. A library of wavelet packets of any scale *s*, frequency *f*, and position *p* is given by:


(3) $$\psi_{sfp}\left(t\right)=2^{-s/2}\psi_{f}\left(2^{-s}t-p\right).$$

The wavelet packets {ψ_*sfp*_ : *p* ∈ *Z*} include a large collection of potential orthonormal bases. An optimal basis can be chosen by the best-basis algorithm (Coifman and Wickerhauser, 1992). Furthermore, an optimal mother wavelet can be chosen by Neretti and Intrator (2002). Following robust statistics methods to prune some of the basis functions, one gets 121 basis functions, which we term brain activity features (BAFs). Based on a given labeled-BAFs dataset, various models can be created for different discriminations of these labels. In the linear case, these models are of the form:


(4) $$V_{k}\left(w,x\right)=\Psi \left(\sum_{i}w_{i}x_{i}\right),$$

where, *w* is a vector of weights and Ψ is a transfer function that can either be linear, e.g., Ψ (*y*) = *y*, or sigmoidal for logistic regression Ψ (*y*) = 1/ (1 + *e*^−*y*^).

From these BAFs, several linear and non-linear combinations were obtained using machine learning techniques on previously collected, labeled datasets. These datasets included data about participants undergoing different cognitive and emotional tasks. Specifically, VC9 was found with linear discriminant analysis technique (LDA, Ye and Li, 2005) on the 121 BAFs to be the best separator between an auditory detection task (higher cognitive load) and an auditory classification task (lower cognitive load, Molcho et al., 2021). VC9 was also recently validated as a cognitive load biomarker using the *n*-back task, auditory detection task, and interruption task (Maimon et al., 2020; Bolton et al., 2021; Molcho et al., 2021). It was shown that VC9 activity increased with increasing levels of cognitive load within cognitively healthy participants (Maimon et al., 2020; Bolton et al., 2021), and showed higher sensitivity than the theta band (Maimon et al., 2021). Neurosteer^®^ provided a sample per second activity of EEG bands (i.e., delta, theta, alpha, beta, and gamma) and VC9.

#### Procedure

The Aurora Electrode strip with three frontal electrodes was attached to each subject’s forehead and connected to the device for brain activity recording. The participants were then asked to complete a standard beginner’s task under the surgical simulator Simbionix LAP MENTOR*™* (Simbionix, Airport City, Israel), which involved grasping and clamping blood vessels using two different laparoscopic arms. The same task was performed by all subjects. At the end of the task, the participants were rated by the surgical simulator based on three main parameters: accuracy in the execution of the required task, economy of movement (to use as few movements as necessary to produce the technical goal), and time required to accomplish the task. All the behavioral parameters were processed by the virtual simulator Simbionix and were presented in graphs and values at the end of the technical goal. All participants were monitored by the EEG device and were given three attempts to perform the same task. Participants performed three consecutive trials in the same session, with a 5-min break between the trials. The performance of each participant was scored by the surgical simulator’s algorithm based on accuracy, economy of movement, and time to complete the task. The concurrent activity of the theta, delta, and VC9 biomarker was extracted by the Aurora system.

#### Statistical Analysis

Behavioral performance was extracted from the surgical simulator after each trial of each participant, including accuracy (in percentages), economy of movement (in percentages), and time to exceed the trial (in seconds). An initial analysis of all bands revealed that the theta band (averaged dBm at 4–7 Hz), the delta band (averaged dBm at 0.5–4 Hz), and VC9 biomarker (normalized between 1 and 100) were the only three EEG features that were both significant in at least one effect of the LMM analysis and one correlation with the repeated correlation analysis, and therefore were included in the analysis (see Supplementary Appendix B for the results of the other oscillations). All features were averaged throughout the task completion. Activity of all the dependent variables was averaged per trial per participant and reported as a mean with a SD. Two main analyses were performed on the behavioral and EEG data: On each experiment, seven mixed linear models (LMM) (Boisgontier and Cheval, 2016) were designed (one for each dependent variable) to measure the differences between the three simulator trials for each dependent variable. The model used trial numbers (1, 2, or 3) as a within-participants variable, and participants were inserted into the random slope. Since each trial number variable included three levels, indicator variables (aka dummy variables) were computed, with Trial 1 as the reference group. Accordingly, two effects resulted from the model: one from the second trial, which represents the significance of difference between the first and second trials, and one from the third trial, which represents the significance of difference between the first and third trials. These effects were extracted straight from the LMM models, with no need for multiple comparisons corrections. For significant fixed effects, we calculated effect sizes according to Westfall et al. (2014) using an analog of Cohen’s *d* (i.e., the expected mean difference divided by the expected variation of an individual observation). Prior to analyses, each dataset for comparison was revised with Shapiro’s test of normal distribution and Levene’s test of equal variance (for all test results, see Supplementary Appendix B). If any were significant, a Wilcoxon nonparametric test was applied instead of LMM. Two-tailed *p* < 0.05 was considered statistically significant. These analyses were performed using Python Statsmodels (Seabold and Perktold, 2010).

The second analysis was designed to evaluate the correlation between individual performance and the EEG feature. We took into this analysis each of the EEG features’ activity and behavioral performance per each participant’s trial. Since during each experiment, each participant underwent three task trials, the data points are not independent. To consider both results from repeated participants’ trials and between participants, we used the repeated measures correlation (rmcorr, Bakdash and Marusich, 2017). Rmcorr estimates the common regression slope, the association shared among individuals. This analysis was underscored by the rmcorr R package (Bakdash and Marusich, 2017), using 1.4.1717 R studio (RStudio Team, 2021). An initial analysis of all bands revealed that the theta band (averaged dBm at 4–7 Hz), the delta band (averaged dBm at 0.5–4 Hz), and VC9 biomarker (normalized between 1 and 100), were the only three EEG features that were both significant in at least one effect of the LMM analysis and one correlation with the repeated correlation analysis (see Supplementary Appendix B). The statistical analyses took into consideration the averaged VC9, delta, and theta activity during each task repetition (an overall average across experiments and trials of 144.17 s).

### Results

#### Behavioral Measurements

**FIGURE 2:**
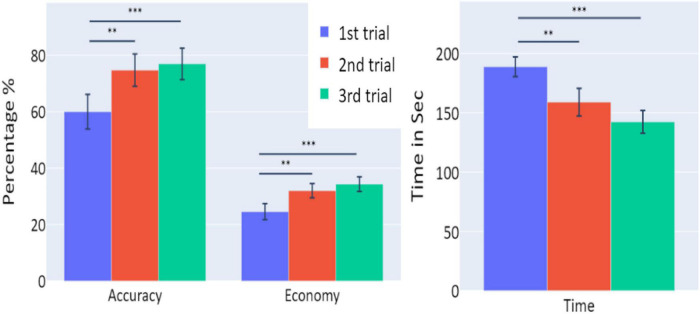
The means of accuracy (in percentage), economy of movement (in percentage), and time to exceed the task (in seconds) obtained in the three trials of Experiment 1, as a function of task repetitions: first trial (blue), second trial (red), and third trial (green), for all participants (*n* = 19). Error bars represent standard errors. *p < 0.05, ^**^p < 0.01, and ^***^p < 0.001.

A full description of the mixed linear models’ (LMM) parameters (coefficients, standard errors, *Z*-scores, *p*-values, and confidence intervals) for all models used in this study are presented in Table 1. Averages and standard errors of behavioral data in Experiment 1 are presented in Figure 2.

**TABLE 1:** Coefficients, standard errors, *Z*-scores, *p*-values, and confidence intervals for all effects depicted in the three LMM analyses applied in the three experiments of the present study.

Experiment	Feature	Effect	Coef.	Std. Err.	z	*p* > |*z*|	0.025	0.975
Experiment 1	Economy	Intercept	25.18	1.931	13.039	<0.001	21.395	28.965
		1st trial vs. 2nd trial	6.82	1.892	3.605	<0.001	3.112	10.528
		1st trial vs. 3rd trial	7.968	1.894	4.206	<0.001	4.255	11.681
	Time	Intercept	185.841	5.33	34.867	<0.001	175.395	196.288
		1st trial vs. 2nd trial	–26.789	9.513	–2.816	0.005	–45.434	–8.144
		1st trial vs. 3rd trial	–40.998	8.638	–4.746	<0.001	–57.929	–24.068
	VC9	Intercept	49.531	1.006	49.215	<0.001	47.558	51.503
		1st trial vs. 2nd trial	–1.506	0.594	–2.537	0.011	–2.67	–0.343
		1st trial vs. 3rd trial	–1.75	0.757	–2.313	0.021	–3.233	–0.267
	Theta	Intercept	–13.324	0.625	–21.314	<0.001	–14.549	–12.099
		1st trial vs. 2nd trial	–0.556	0.407	–1.367	0.172	–1.354	0.241
		1st trial vs. 3rd trial	–0.397	0.589	–0.675	0.5	–1.551	0.756
	Delta	Intercept	–2.907	0.742	–3.919	0.000	–4.360	–1.453
		1st trial vs. 2nd trial	–1.493	0.500	–2.989	0.003	–2.472	–0.514
		1st trial vs. 3rd trial	–1.100	0.608	–1.809	0.070	–2.292	0.092
Experiment 2	Economy	Intercept	39.225	2.637	14.873	<0.001	34.056	44.394
		1st trial vs. 2nd trial	–0.525	3.49	–0.15	0.88	–7.365	6.316
		1st trial vs. 3rd trial	4.375	3.412	1.282	0.2	–2.312	11.062
	Time	Intercept	119.596	11.414	10.478	<0.001	97.224	141.968
		1st trial vs. 2nd trial	–8.396	12.449	–0.674	0.5	–32.796	16.004
		1st trial vs. 3rd trial	–22.883	12.971	–1.764	0.078	–48.306	2.54
	VC9	Intercept	49.451	1.341	36.874	<0.001	46.823	52.079
		1st trial vs. 2nd trial	–0.466	0.726	–0.642	0.521	–1.889	0.957
		1st trial vs. 3rd trial	–0.758	0.966	–0.784	0.433	–2.652	1.136
	Theta	Intercept	–13.218	0.824	–16.043	<0.001	–14.833	–11.603
		1st trial vs. 2nd trial	–0.433	0.611	–0.709	0.478	–1.63	0.764
		1st trial vs. 3rd trial	–0.357	0.638	–0.56	0.576	–1.608	0.894
	Delta	Intercept	–2.117	0.953	–2.221	0.026	–3.986	–0.249
		1st trial vs. 2nd trial	–0.123	0.901	–0.137	0.891	–1.888	1.642
		1st trial vs. 3rd trial	–0.862	0.968	–0.891	0.373	–2.759	1.035
Experiment 3	Accuracy	Intercept	59.067	4.008	14.738	<0.001	51.211	66.922
		1st trial vs. 2nd trial	13.023	3.715	3.505	<0.001	5.741	20.305
		1st trial vs. 3rd trial	21.607	3.887	5.559	<0.001	13.99	29.225
	Economy	Intercept	22.003	1.845	11.923	<0.001	18.386	25.62
		1st trial vs. 2nd trial	14.209	1.677	8.47	<0.001	10.921	17.497
		1st trial vs. 3rd trial	17.665	2.305	7.664	<0.001	13.147	22.183
	Time	Intercept	182.022	4.539	40.103	<0.001	173.127	190.918
		1st trial vs. 2nd trial	–31.743	7.613	–4.17	<0.001	–46.664	–16.822
		1st trial vs. 3rd trial	–58.707	5.368	–10.936	<0.001	–69.228	–48.185
	VC9	Intercept	54.489	1.633	33.375	<0.001	51.289	57.689
		1st trial vs. 2nd trial	1.915	1.002	1.911	0.056	–0.049	3.88
		1st trial vs. 3rd trial	2.493	1.967	1.267	0.205	–1.362	6.348
	Theta	Intercept	–6.473	0.899	–7.197	<0.001	–8.235	–4.71
		1st trial vs. 2nd trial	1.285	0.548	2.346	0.019	0.211	2.358
		1st trial vs. 3rd trial	1.432	1.034	1.385	0.166	–0.594	3.459
	Delta	Intercept	5.950	1.438	4.138	0.000	3.132	8.768
		1st trial vs. 2nd trial	2.174	1.157	1.879	0.060	–0.094	4.441
		1st trial vs. 3rd trial	3.609	1.878	1.922	0.055	–0.072	7.289

*All LMMs included trial (1/2/3) as a within-participants variable, with encoded effects of Trial 1 vs. Trial 2 and Trial 1 vs. Trial 3.*

A significant increase of the participants’ accuracy was observed between the first and second trials and between the first and third trials (*W* = 16, *p* = 0.004; and *W* = 10, *p* = 0.004, respectively), as well as a significant increase in the economy of movement (*p* < 0.001, *d* = −0.799; *p* < 0.001, *d* = −1.054, respectively). The average time required for the completion of the task was also significantly reduced between the first and second trials, and first and third trials (*p* = 0.005, *d* = 0.854; *p* < 0.001, *d* = 1.562, respectively).

#### Electroencephalography Features

**FIGURE 3:**
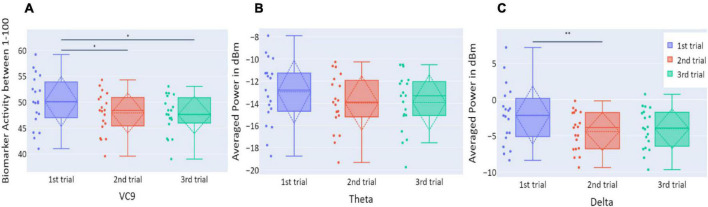
**(A)** The distribution of participant VC9 activity (normalized between 1 and 100); **(B)** the distribution of theta (averaged power of 4–7 Hz in dBm); and **(C)** the distribution of delta (averaged power of 0.5–4 Hz in dBm), obtained in the three trials of Experiment 1, as a function of task repetitions: first trial (blue), second trial (red), and third trial (green), for all participants (*n* = 19). Dashed lines represent means and SDs. *p < 0.05, ^**^p < 0.01, and ^***^p < 0.001.

Distributions of VC9, theta, and delta data in Experiment 1 are presented in Figure 3. VC9 activity was significantly reduced between the first and second attempts and between the first and third attempts (*p* = 0.011, *d* = 0.474; *p* = 0.021, *d* = 0.503, respectively). Delta activity significantly decreased between the first and second attempts (*p* = 0.003, *d* = 0.625). Theta did not exhibit any significant differences between the trials (all *p*’s > 0.05).

#### Correlations Between Electroencephalography Features Activity and Behavioral Performance

**FIGURE 4:**
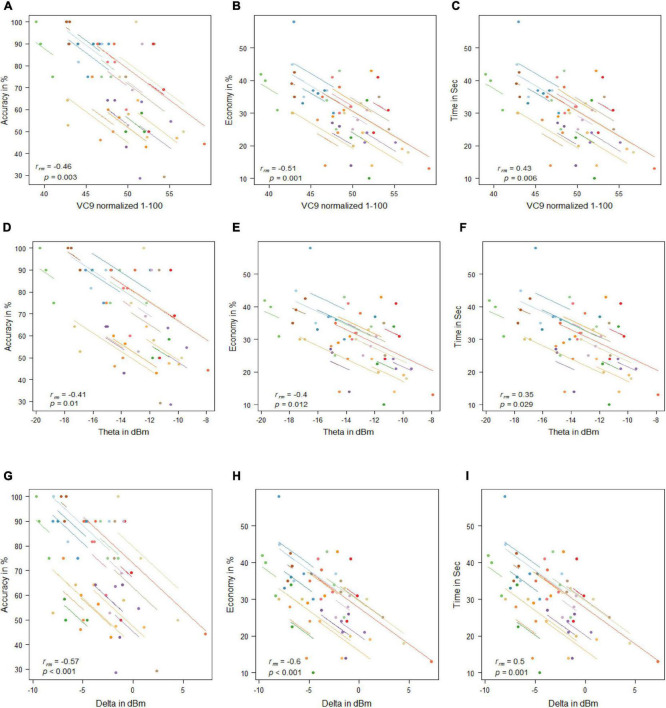
Mean activity of VC9 as a function of accuracy **(A)**, economy **(B)**, and time **(C)**; theta as a function of accuracy **(D)**, economy **(E)**, and time **(F)**; and delta as a function of accuracy **(G)**, economy **(H)**, and time **(I)** per trial and participant obtained in Experiment 1 (*n* = 19). R_rm_ and *p-*values presented.

VC9 activity significantly correlated with all three behavioral measurements and was found to decrease with better performance (i.e., negatively correlated with accuracy and economy of movement and positively correlated with time): *r*_*rm*_ = −0.46, *p* = 0.003; *r*_*rm*_ = −0.51, *p* = 0.001; and *r*_*rm*_ = 0.43, *p* = 0.006 for accuracy, economy of movement, and time, respectively; see Figure 4. Delta also significantly correlated with all three measurements: *r*_*rm*_ = −0.57, *p* < 0.001; *r*_*rm*_ = −0.6, *p* < 0.001; and *r*_*rm*_ = 0.5, *p* = 0.001 for accuracy, economy of movement, and time, respectively. Theta decreased with higher accuracy and economy of movement and increased with time: *r*_*rm*_ = −0.41, *p* = 0.01; *r*_*rm*_ = −0.4, *p* = 0.012; and *r*_*rm*_ = 0.35, *p* = 0.029 for accuracy, economy of movement, and time, respectively.

### Discussion

Experiment 1 results exhibited a significant improvement in all behavioral measurements of performance, as well as a significant decrease in VC9 and delta activity (but not theta) between the three trials. In addition, VC9 and delta activity decreased with better individual performance, which was expressed by the significant correlations between all three behavioral measures and VC9 and delta activity. Theta also exhibited individual differences and decreased with higher accuracy and economy of movement, and marginally increased with longer time to complete the task. These results suggest that changes in cognitive load correspond to performance in a surgery simulator as shown by VC9, delta, and, to some extent, frontal theta.

Next, we aimed to explore the effect of nighttime sleep memory consolidation on task performance in the simulator and participants’ brain activity. Hence, we conducted a second experiment that included 10 participants who had also participated in Experiment 1. They performed an additional three-trial session on the consecutive day.

## Experiment 2

### Materials and Methods

#### Participants and Procedure

On the day following Experiment 1, 10 randomly chosen participants performed an additional three trials of the same procedure.

### Results

#### Behavioral Measurements

**FIGURE 5:**
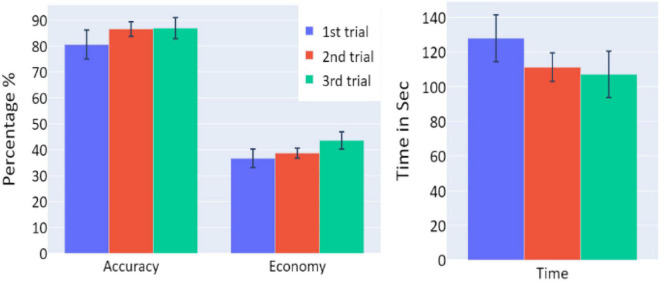
The means of accuracy (in percentage), economy of movement (in percentage), and time to exceed the task (in seconds) obtained in the three trials of Experiment 2, as a function of task repetitions: first trial (blue), second trial (red), and third trial (green), for participants who underwent Experiment 2 (*n* = 10). Error bars represent standard errors. *p < 0.05, ^**^p < 0.01, and ^***^p < 0.001.

Behavioral performance did not differ between the three trials of this session in any of the measurements (all *p*’s > 0.05; see Figure 5).

#### Electroencephalography Features

**FIGURE 6:**
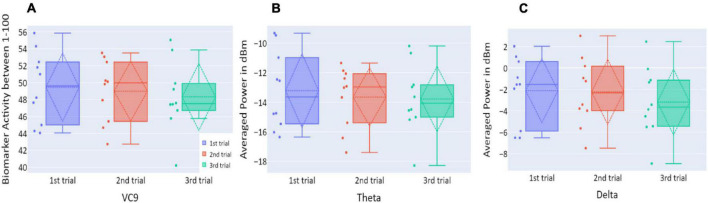
**(A)** The distribution of user VC9 activity (normalized between 1 and 100); **(B)** the distribution of theta (averaged power of 4–7 Hz in dBm); and **(C)** the distribution of delta (averaged power of 0.5–4 Hz in dBm), obtained in the three trials of Experiment 2, as a function of task repetitions: first trial (blue), second trial (red), and third trial (green), for all participants (*n* = 10). Dashed lines represent means and SDs. *p < 0.05, ^**^p < 0.01, and ^***^p < 0.001.

Activity levels of all EEG features (VC9, theta, and delta) did not differ between the trials of the session (*p* > 0.05; see Figure 6).

#### Correlations Between Electroencephalography Features Activity and Behavioral Performance

**FIGURE 7:**
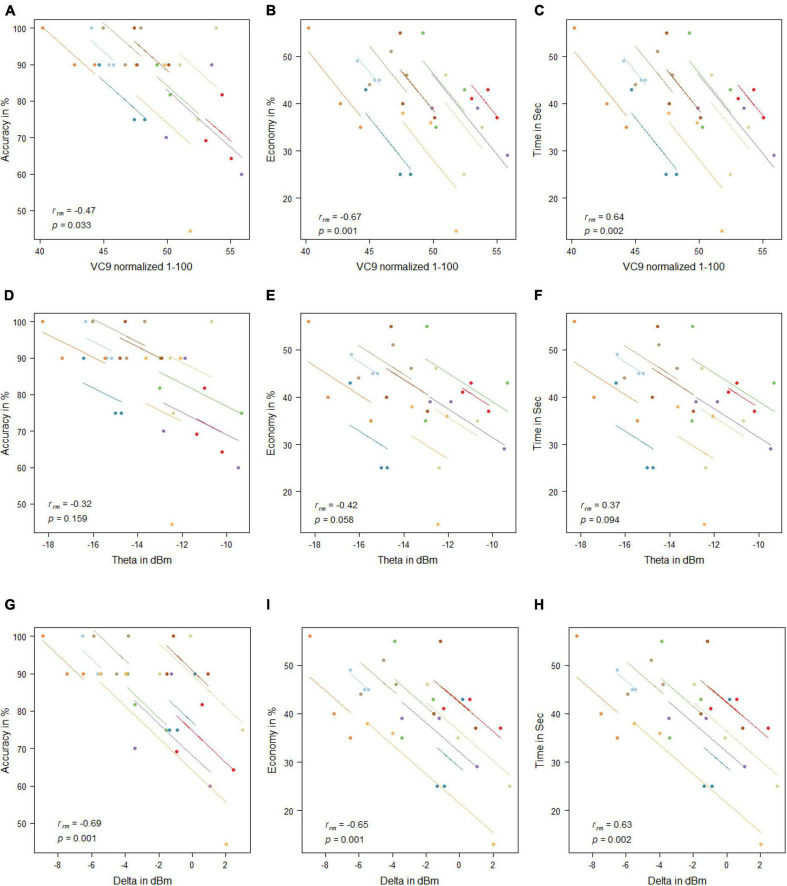
Mean activity of VC9 as function of accuracy **(A)**, economy **(B)**, and time **(C)**; theta as function of accuracy **(D)**, economy **(E)**, and time **(F)**; and delta as function of accuracy **(G)**, economy **(H)**, and time **(I)** per trial and participant obtained in Experiment 2 (*n* = 10). R_rm_ and *p-*values presented.

VC9 activity significantly correlated with all three behavioral measurements and was found to decrease with better participant performance (i.e., negatively correlated with accuracy and economy of movement and positively correlated with time): *r*_*rm*_ = −0.47, *p* = 0.033; *r*_*rm*_ = −0.67, *p* = 0.001; and *r*_*rm*_ = 0.64, *p* = 0.002 for accuracy, economy of movement, and time, respectively. Delta also significantly correlated with all three measurements: *r*_*rm*_ = −0.69, *p* = 0.001; *r*_*rm*_ = −0.65, *p* = 0.001; and *r*_*rm*_ = 0.63, *p* = 0.002 for accuracy, economy of movement, and time, respectively. Theta did not correlate significantly with any of the behavioral measurements (all *p*’s > 0.05; see Figure 7).

#### Comparison Between Experiment 1 and Experiment 2

**FIGURE 8:**
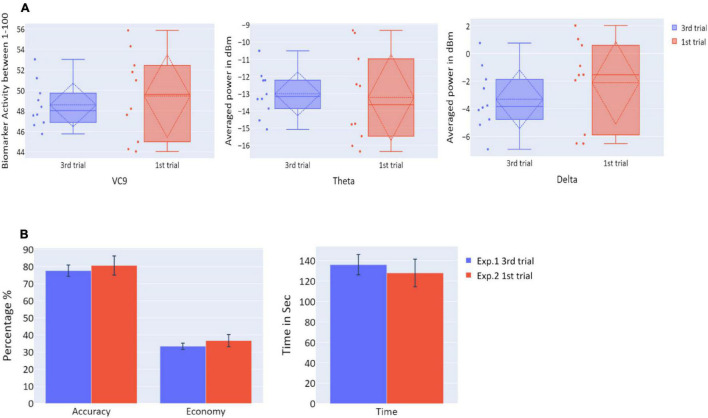
**(A)** The distribution of VC9 (normalized between 1 and 100), theta (averaged power of 4–7 Hz in dBm), and delta (averaged power of 0.5–4 Hz in dBm), obtained in the last trial repetition of Experiment 1 (blue), and the first task repetition of Experiment 2 (red), for participants who participated in both Experiment 1 and Experiment 2 (*n* = 10). Dashed lines represent means and SDs. **(B)** The means of accuracy (in percentage), economy of movement (in percentage), and time to exceed the task (in seconds), obtained in the last trial repetition of Experiment 1 (blue), and the first task repetition of Experiment 2 (red), for participants who participated in both Experiment 1 and Experiment 2 (*n* = 10). Error bars represent standard errors.

To explore the “offline gains” between Experiment 1 and Experiment 2, behavioral performance and brain activity of the 10 participants who participated in both experiments were studied. Paired *t*-tests on the activity of the 10 participants who underwent both experiments were calculated. The *t*-tests compared accuracy, economy of movement, time, VC9, theta, and delta activity for the last trial of Experiment 1 and the first trial of Experiment 2. Both behavioral performance and brain activity were the same in the first trial of Experiment 2 relative to the last trial of Experiment 1: all *p*’s > 0.05; see Figure 8 and Table 2.

**TABLE 2:** *t*/*W* statistics and *p*-values for *t*-tests/Wilcoxon tests comparing Experiment 1 last trial and Experiment 2 first trial, for participants who participated in both Experiment 1 and Experiment 2 (*n* = 10).

Feature	Statistic	*p*-value
Accuracy	*t* = −0.476	0.645
Economy	*t* = −0.831	0.428
Time	*t* = 0.524	0.613
VC9	*W* = 21	0.557
Theta	*W* = 26	0.922
Delta	*t* = 1.103	0.298

### Discussion

The results of Experiment 2 exhibited a clear correspondence between behavioral performance and EEG features. First, both behavioral performance and all EEG features’ activity did not exhibit significant differences between the three trials, meaning that participants’ performance did not improve, and theta, delta, and VC9 activity did not decrease between the three trials. Second, VC9 and delta activity decreased with better behavioral performance, as depicted by a significant correlation with all three behavioral measurements. Theta correlated with accuracy but not with economy of movement and time.

The comparison between the last trial of Experiment 1 and the first trial of Experiment 2 showed no difference between the two in any of the behavioral or neurophysiological measures. However, taken together with the null improvement during these three sessions, this lack of difference may be explained by participants having reached their asymptotic level (ceiling effect): that participants reached their maximum ability on the third trial of the first day, and maintained it through the first trials of the second day. To further reveal “offline gains” from nighttime sleep consolidation, we conducted a third experiment on an additional 19 medical students who had not participated in Experiments 1 and 2. Participants performed a single-task trial per day for three consecutive days. This was done to make sure participants did not reach asymptotic performance levels before the nighttime sleep consolidation, since they would only complete a single trial before nighttime sleep.

## Experiment 3

### Materials and Methods

#### Participants and Procedure

Nineteen (63% females) healthy medical students from their first to sixth years of studying (mean = 4), who did not participate in former Experiments 1 and 2, with mean age of 25.631 (SD = 2.532), participated in Experiment 3. All students had no prior experience using a surgical simulator. Participants underwent the same task as in Experiments 1 and 2, with one trial per day over three consecutive days.

### Results

#### Behavioral Measurements

**FIGURE 9:**
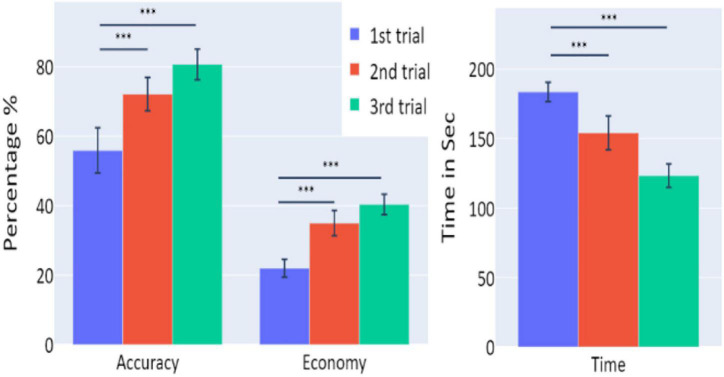
The means of accuracy (in percentage), economy of movement (in percentage), and time to exceed the task (in seconds) obtained in the three trials of Experiment 3, as a function of task repetitions: first trial (blue), second trial (red), and third trial (green), for participants who underwent Experiment 3 (*n* = 19). Error bars represent standard errors. *p < 0.05, ^**^p < 0.01, and ^***^p < 0.001.

Averages and standard errors of the behavioral data in Experiment 3 are presented in Figure 9. A significant increase of the participants’ accuracy was observed between the first and second trials and between the first and third trials (*W* = 11, *p* = 0.002; *W* = 2, *p* < 0.001, respectively), as well as a significant increase in the economy of movement (*p* < 0.001, *d* = −1.762; *p* < 0.001, *d* = −1.83, respectively). The average time required for the completion of the task was also significantly reduced between the trials (*p* < 0.001, *d* = 1.014; *p* < 0.001, *d* = 3.171 for first vs. second trials and first vs. third trials, respectively).

#### Electroencephalography Features

**FIGURE 10:**
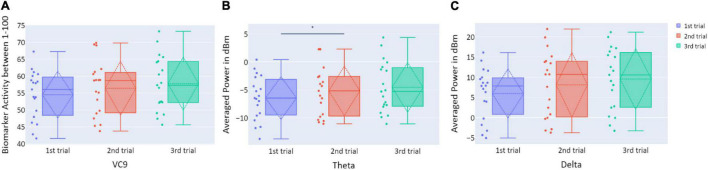
**(A)** The distribution of user VC9 activity (normalized between 1 and 100); **(B)** the distribution of theta (averaged power of 4–7 Hz in dBm); and **(C)** the distribution of delta (averaged power of 0.5–4 Hz in dBm), obtained in the three trials of Experiment 3, as a function of task repetitions: first trial (blue), second trial (red), and third trial (green), for all participants (*n* = 19). Dashed lines represent means and SDs. *p < 0.05, ^**^p < 0.01, and ^***^p < 0.001.

Distributions of VC9, theta, and delta data in Experiment 3 are presented in Figure 10. There were no significant differences in VC9 and delta activity between the trials on consecutive days (all *p*’s > 0.05). Theta exhibited a significant difference between the first and second trials (*p* = 0.019, *d* = −0.354), but the difference between the first and third trials did not reach significance level (*p* > 0.05).

#### Correlations Between Electroencephalography Features Activity and Behavioral Performance

**FIGURE 11:**
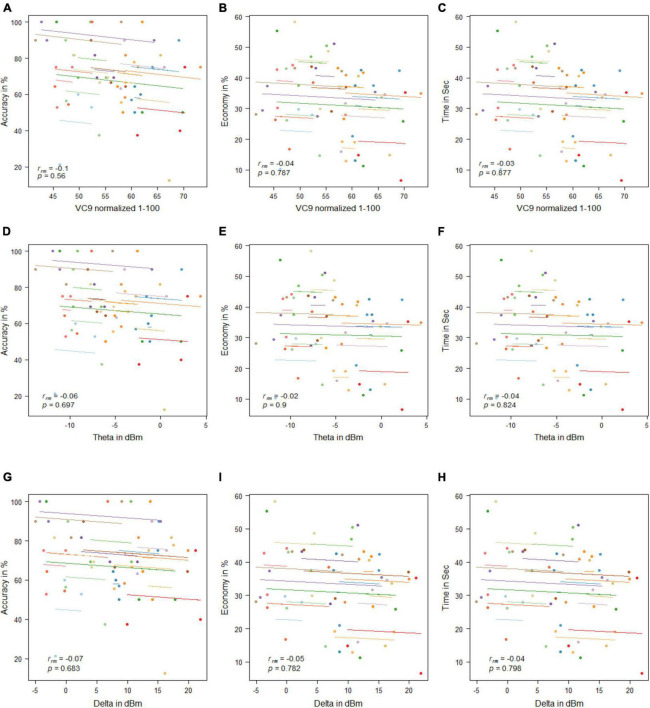
Mean activity of VC9 as a function of accuracy **(A)**, economy **(B)**, and time **(C)**; theta as a function of accuracy **(D)**, economy **(E)**, and time **(F)**; and delta as a function of accuracy **(G)**, economy **(H)**, and time **(I)** per trial and participant obtained in Experiment 3 (*n* = 19). R_rm_ and *p-*values presented.

No significant correlations were found between any of the EEG features and behavioral measurement (all *p*’s > 0.05; see Figure 11).

### Discussion

Experiment 3 results reflect “offline gains”: participants’ performances under the simulator improved with the trials’ repetition, although each occurred on the consecutive day. However, VC9, delta, and theta did not show significant differences between the trials, nor did they exhibit significant correlations with any of the behavioral measurements. This discrepancy might be explained by the different brain networks that take part in “online” versus “offline” learning; see general discussion below.

## General Discussion

In this study, we continuously measured cognitive load levels using theta and band power and VC9 biomarker activity during task performance on a surgical simulator. A single-channel EEG device utilizing decomposition of the EEG signal *via* harmonic analysis was used to obtain the novel VC9 biomarker as well as the frequently used theta and delta bands. All EEG features (i.e., theta, delta, and VC9) were previously shown to correlate with cognitive load levels (Antonenko et al., 2010; Maimon et al., 2020; Bolton et al., 2021; Molcho et al., 2021). Experiment 1 consisted of three repeating trials on the surgery simulator, and its results showed that participants’ performances improved while VC9 and delta activity decreased. Additionally, VC9, delta, and, to some extent, theta band decreased with higher individual performance. Overall, these results support previous findings that as the participant becomes more proficient in performing a task, as depicted by the behavioral performance, the pre-frontal activity associated with cognitive load is reduced (Takeuchi et al., 2013).

To examine the effect of nighttime sleep memory consolidation, we conducted Experiment 2, which included three simulator trials on the consecutive day. Testing the “offline gains” refers to the improvements in skill acquisition preceding a consolidation during nighttime sleep between tasks trainings and without further practice (Walker et al., 2002). Results revealed no significant differences in performance or EEG features between the three trials. Although they did not exhibit significant decreases between the trials, the VC9, delta, and, to some extent, theta still exhibited significant correlations with individual performances. Further analysis revealed that both behavioral and EEG features results maintained the same levels between the last trial of the first day and the first trial on the second day. Taken together, these findings potentially indicate that “offline gains” were not detected, since participants reached their maximum performance levels on the last trial of the first day and maintained them throughout the trials on the following day.

Consequently, we conducted an additional experiment in which 19 medical students performed a single simulator trial on three consecutive days. This was done to prevent participants from reaching their maximum performance levels on the first day, and to reveal the nighttime sleep memory consolidation related to “offline gains.” Indeed, participants’ performances in all behavioral measurements improved significantly between the testing days. Interestingly, VC9 and delta showed no difference between the trials, and the theta band even showed a mild increase in activity between the trials. This lack of increase in activity can be explained by the difference in brain networks that are involved in fast vs. slow stages of motor skill learning (Dayan and Cohen, 2011). Similar to online learning, fast motor skill acquisition occurs during task training and could last minutes (Karni et al., 1995). In the slow stage, further gains are achieved across multiple sessions of training, mostly divided by nighttime sleep consolidation. The neural substrates during the fast stage show complex brain activation patterns. First, they include increasing activity in the Supplementary Motor Area (SMA), dorsomedial striatum (DMS), premotor cortex (PM), posterior parietal cortex (PPC), and posterior cerebellum (Honda et al., 1998; Grafton et al., 2002; Floyer-Lea and Matthews, 2005). This reflects the requirement of additional cortical brain activity during practice. At the same time, the fast stage also causes decreased activity of the dorsolateral prefrontal cortex (DLPFC), primary motor cortex (M1), and pre Supplementary Motor Area (preSMA, Poldrack, 2000). These decreases may suggest that with online practice, one uses fewer neuronal resources (Hikosaka et al., 2002). Conversely, the slow motor skill stage is characterized by increased activity in M1, S1, SMA, and DLS (Floyer-Lea and Matthews, 2005), and decreased activity in the lateral cerebellum (Lehéricy et al., 2005). Notably, the decrease in the frontal area (DLPFC), which commonly correlates with WM load (Manoach et al., 1997), is not visible during this slow learning stage. Thus, progress from fast to slow stages can be generalized to the shift from anterior to more posterior brain regions (Floyer-Lea and Matthews, 2005).

The current WM biomarkers that were used in the present research were extracted *via* frontal single-EEG channel, and although this electrode can extract additional brain activity beyond the frontal lobe, we aimed to focus on biomarkers that reflect frontal activity associated with WM load. Taken together, this may explain the results obtained in Experiment 3 of the present study, which revealed prominent “offline gains” within the behavioral performance of the task, but no decrease within the current EEG WM load biomarkers. Further research, however, should look for novel biomarkers that will be able to correlate with such posterior/limbic activity. Finally, this discrepancy may further support frontal theta, delta, and VC9 as continuous measurements of cognitive load, to monitor WM load during task completion and not as evaluators of cognitive load between tasks’ sessions. Therefore, these biomarkers may be adequate for assessing laparoscopic dexterity of non-experts during their first real-life surgeries. However, for the assessment of surgeons’ improvement in between laparoscopic practices, other biomarkers should be considered.

The activation patterns obtained in Experiments 1 and 2 are compatible with WM load, as previously described (Zarjam et al., 2011; Wang et al., 2016). Delta and VC9 activity decreased with the lower cognitive load that resulted from simulator trial repetition, but the theta band activity did not decrease. As shown in previous studies (Maimon et al., 2020; Molcho et al., 2021), VC9 was found to be more sensitive to lower loads than theta and delta, as its activity also showed a significant decrease between the first and third task trials. This further validates VC9 as an effective biological measurement for the assessment of cognitive load while performing laparoscopic tasks using the surgical simulator specifically and suggests that it may provide a measure of cognitive functioning during surgery. Due to the unobtrusive nature of mobile EEG devices, such devices can be used by surgeons during live operations. Intraoperative evaluation can provide an objective metric to assess surgeons’ performance in real-life scenarios and measure additional unobservable behaviors, such as mental readiness (Cha and Yu, 2021).

Since simulation-based training is an effective tool for acquiring practical skills, the question remains as to which methods should be utilized to optimize this process and achieve better assessment of improved manual dexterity. Specifically, it is not fully understood which brain processes during simulation-based training translate to better skill acquisition through practice. Here, we show that VC9 and delta, collected *via* single-channel EEG set, can be reliably used to monitor and assess participants’ WM levels during manual practice. Furthermore, the correlations between VC9, delta, and improved simulator scores can be translated to other areas and used in other procedures that require manual training. Importantly, as this device is portable and can be easily worn inside the operating room, it could potentially be used to predict participants’ WM loads and indirect performance during real-life operating procedures.

This study has several limitations. The analyses were performed on young medical internists, which may not accurately reflect the overall medical population. Additionally, evaluating finer differences between the experiments requires a larger cohort of participants. Future studies on a larger and more diverse population should further validate the findings presented in this work, as well as study the effect of prolonged breaks on the activity of these biomarkers. Moreover, a 24-h break period between the sessions may not be sufficient to study the effects of long-term memory on the prefrontal brain activity during the simulation. Future studies should evaluate longer time windows to assess the temporal changes in the activity of the working-memory-associated biomarkers and search for additional biomarkers to represent other cerebral regions beyond the frontal regions (like the limbic or motor systems).

To conclude, in this study, we extracted the previously validated cognitive load biomarkers—theta, delta, and VC9—from a novel single-channel EEG using advanced signal analysis. Results showed high correlations between the EEG features and participants’ individual performances using a surgical simulator. As surgical simulations allow doctors to gain important skills and experience needed to perform procedures without any patient risk, evaluation and optimization of these effects on medical staff are crucial. This could potentially be expanded to evaluate the efficacy of different medical simulations to provide more efficient training to medical staff and to measure cognitive and mental loads in real laparoscopic surgeries.

## Data Availability Statement

The datasets presented in this article are not readily available because the data that support the findings of this study are available for simbionix and Neurosteer Ltd. Restrictions apply to the availability of these data, which were used under license for this study. Requests to access the datasets should be directed to lior@neurosteer.com.

## Ethics Statement

The studies involving human participants were reviewed and approved by Galilee Medical Center Institutional Review Board. The patients/participants provided their written informed consent to participate in this study.

## Author Contributions

AB, MB, NM, and NI conceived and planned the experiments. AB, MB, and DD carried out the experiments. NI and LM designed and provided tech support for the EEG device. MB, NM, and LM contributed to the interpretation of the results. MB and NM took the lead in writing the manuscript. All authors provided critical feedback and helped shape the research, analysis, and manuscript.

## Conflict of Interest

NM is a part-time researcher at Neurosteer Ltd., which supplied the EEG system. LM is a clinical researcher at Neurosteer Ltd. NI is the founder of Neurosteer Ltd. The remaining authors declare that the research was conducted in the absence of any commercial or financial relationships that could be construed as a potential conflict of interest.

## Publisher’s Note

All claims expressed in this article are solely those of the authors and do not necessarily represent those of their affiliated organizations, or those of the publisher, the editors and the reviewers. Any product that may be evaluated in this article, or claim that may be made by its manufacturer, is not guaranteed or endorsed by the publisher.
