# Supplementary material for: Continuous Monitoring of Mental Load During Virtual Simulator Training for Laparoscopic Surgery Reflects Laparoscopic Dexterity: A Comparative Study Using a Novel Wireless Device
Source: Front Neurosci. 2022 Jan 20;15:694010. doi: 10.3389/fnins.2021.694010 (PMC8811150; doi:10.3389/fnins.2021.694010)
Supplement: Supplementary file 2 [file Data_Sheet_2.PDF]

Appendix B:

Experiment	***Feature***	***Effect***	*Coef.*	*Std.Err.*	*z*	*p>/z/*	*[0.025*	*0.975]*
Experiment 1	Alpha	*Intercept*	-17.732	0.511	-34.71	0	-18.733	-16.731
		1st trial vs. 2nd trial	-0.36	0.296	-1.215	0.224	-0.94	0.22
		1st trial vs. 3rd trial	-0.222	0.386	-0.574	0.566	-0.977	0.534
	Beta	*Intercept*	-19.945	0.59	-33.812	0	-21.101	-18.789
		1st trial vs. 2nd trial	-0.331	0.278	-1.189	0.234	-0.877	0.215
		1st trial vs. 3rd trial	-0.39	0.261	-1.492	0.136	-0.902	0.122
	Gamma	*Intercept*	-19.974	0.683	-29.231	0	-21.314	-18.635
		1st trial vs. 2nd trial	-0.392	0.352	-1.114	0.265	-1.083	0.298
		1st trial vs. 3rd trial	-0.709	0.299	-2.37	0.018	-1.295	-0.123
Experiment 2	Alpha	*Intercept*	-18.143	0.731	-24.824	0	-19.575	-16.71
		1st trial vs. 2nd trial	-0.125	0.285	-0.438	0.661	-0.684	0.434
		1st trial vs. 3rd trial	-0.367	0.285	-1.287	0.198	-0.925	0.192
	Beta	*Intercept*	-19.746	1.107	-17.833	0	-21.916	-17.576
		1st trial vs. 2nd trial	-0.771	0.427	-1.807	0.071	-1.608	0.065
		1st trial vs. 3rd trial	-0.734	0.681	-1.077	0.281	-2.07	0.602
	Gamma	*Intercept*	-20.671	1.24	-16.671	0	-23.101	-18.241
		1st trial vs. 2nd trial	-0.684	0.464	-1.474	0.14	-1.594	0.225
		1st trial vs. 3rd trial	-0.649	0.782	-0.829	0.407	-2.182	0.884
Experiment 3	Alpha	*Intercept*	-11.271	0.751	-15.002	0	-12.743	-9.798
		1st trial vs. 2nd trial	1.429	0.493	2.897	0.004	0.462	2.395
		1st trial vs. 3rd trial	1.49	0.806	1.848	0.065	-0.091	3.07
	Beta	*Intercept*	-13.154	0.829	-15.858	0	-14.779	-11.528
		1st trial vs. 2nd trial	1.168	0.525	2.225	0.026	0.139	2.197
		1st trial vs. 3rd trial	1.376	0.686	2.005	0.045	0.031	2.721
	Gamma	*Intercept*	-14.379	0.946	-15.203	0	-16.232	-12.525
		1st trial vs. 2nd trial	0.407	0.417	0.976	0.329	-0.41	1.224
		1st trial vs. 3rd trial	0.525	0.569	0.923	0.356	-0.59	1.64

Table 1: LMM results of alpha, beta and gamma oscillations.

Experiment	***EEG Feature***	***Behavioral measurement***	R_{rm}	*p value*
Experiment 1	Alpha	*Accuracy*	-0.16	0.326
		Economy	-0.25	0.127
		Time	0.17	0.313
	Beta	*Accuracy*	-0.2	0.223
		Economy	-0.15	0.363
		Time	0.18	0.28
	Gamma	*Accuracy*	-0.23	0.158
		Economy	0.13	0.414
		Time	0.18	0.276
Experiment 2	Alpha	*Accuracy*	-0.35	0.118
		Economy	-0.32	0.164
		Time	0.27	0.235
	Beta	*Accuracy*	-0.42	0.058
		Economy	-0.36	0.113
		Time	0.43	0.054
	Gamma	*Accuracy*	-0.37	0.095
		Economy	-0.39	0.082
		Time	0.41	0.051
Experiment 3	Alpha	*Accuracy*	-0.02	0.921
		Economy	0	0.992
		Time	-0.06	0.716
	Beta	*Accuracy*	0.01	0.95
		Economy	0.04	0.825
		Time	-0.02	0.905
	Gamma	*Accuracy*	-0.11	0.488
		Economy	-0.11	0.505
		Time	0.08	0.633

Table 2: repeated measures correlations between alpha, beta and gamma oscillations and the individual behavioral performance.

Experiment	Test	Feature	Statistic	*p* values
Experiment 1	Shapiro test for normality	Accuracy	0.937	0.005
		Economy	0.983	0.589
		Time	0.993	0.986
		Delta	0.966	0.113
		Theta	0.982	0.550
		VC9	0.989	0.899
	Levens test for equal variance<br>1st trial vs. 3rd trial	Accuracy	0.061	0.806
		Economy	0.425	0.518
		Time	0.805	0.376
		Delta	1.529	0.224
		Theta	1.515	0.226
		VC9	0.457	0.503
Experiment 2	Shapiro test for normality	Accuracy	0.862	0.001
		Economy	0.956	0.238
		Time	0.880	0.003
		Delta	0.975	0.679
		Theta	0.984	0.928
		VC9	0.982	0.866
	Levens test for equal variance<br>1st trial vs. 3rd trial	Accuracy	0.808	0.380
		Economy	0.024	0.878
		Delta	0.013	0.910
		Time	0.043	0.838
		Theta	0.516	0.482
		VC9	0.775	0.390
Experiment 3	Shapiro test for normality	Accuracy	0.962	0.071
		Economy	0.972	0.201
		Time	0.969	0.151
		Delta	2.206	0.146
		Theta	0.265	0.610
		VC9	0.582	0.451
	Levens test for equal variance<br>1st trial vs. 3rd trial	Accuracy	0.808	0.380
		Economy	0.024	0.878
		Time	0.043	0.838
		Delta	1.442	0.238

		Theta	0.516	0.482
		VC9	0.775	0.390
Experiment 1 vs. 2	Shapiro test for normality	Accuracy	0.925	0.125
		Economy	0.962	0.586
		Time	0.944	0.280
		Delta	0.956	0.459
		Theta	0.966	0.661
		VC9	0.967	0.688
	Levens test for equal variance exp. 1 last trial vs. exp. 2 first trial	Accuracy	1.616	0.220
		Economy	1.460	0.243
		Time	0.253	0.621
		Delta	0.778	0.389
		Theta	6.664	0.019
		VC9	7.135	0.016

Table 3: Shapiro tests for normal distribution and Levens tests for equal variance for all comparisons conducted in the present study.
